# Early identification of macrophage activation syndrome secondary to systemic lupus erythematosus with machine learning

**DOI:** 10.1186/s13075-024-03330-9

**Published:** 2024-05-09

**Authors:** Wenxun Lin, Xi Xie, Zhijun Luo, Xiaoqi Chen, Heng Cao, Xun Fang, You Song, Xujing Yuan, Xiaojing Liu, Rong Du

**Affiliations:** 1 Department of Rheumatology, Union hospital, Tongji Medical College, Huazhong University of Science and Technology, Wuhan, China; 2 Department of Rheumatology and Immunology, the Second Xiangya Hospital, Central South University, Changsha, Hunan P.R. China; 3 Clinical Medical Research Center for Systemic Autoimmune Diseases in Hunan Province, Changsha, Hunan P.R. China; 4 Department of Rheumatology, Zhongnan Hospital of Wuhan University, Wuhan, China; 5 Department of Rheumatology, The First Affiliated Hospital, College of Medicine, Zhejiang University, Hangzhou, China; 6 Department of Rheumatology, Tongji Medical College, The Central Hospital of Wuhan, Huazhong University of Science and Technology, Wuhan, China

**Keywords:** Machine learning, Systemic lupus erythematosus, Macrophage activation syndrome

## Abstract

**Objective:**

The macrophage activation syndrome (MAS) secondary to systemic lupus erythematosus (SLE) is a severe and life-threatening complication. Early diagnosis of MAS is particularly challenging. In this study, machine learning models and diagnostic scoring card were developed to aid in clinical decision-making using clinical characteristics.

**Methods:**

We retrospectively collected clinical data from 188 patients with either SLE or the MAS secondary to SLE. 13 significant clinical predictor variables were filtered out using the Least Absolute Shrinkage and Selection Operator (LASSO). These variables were subsequently utilized as inputs in five machine learning models. The performance of the models was evaluated using the area under the receiver operating characteristic curve (ROC-AUC), F1 score, and F2 score. To enhance clinical usability, we developed a diagnostic scoring card based on logistic regression (LR) analysis and Chi-Square binning, establishing probability thresholds and stratification for the card. Additionally, this study collected data from four other domestic hospitals for external validation.

**Results:**

Among all the machine learning models, the LR model demonstrates the highest level of performance in internal validation, achieving a ROC-AUC of 0.998, an F1 score of 0.96, and an F2 score of 0.952. The score card we constructed identifies the probability threshold at a score of 49, achieving a ROC-AUC of 0.994 and an F2 score of 0.936. The score results were categorized into five groups based on diagnostic probability: extremely low (below 5%), low (5–25%), normal (25–75%), high (75–95%), and extremely high (above 95%). During external validation, the performance evaluation revealed that the Support Vector Machine (SVM) model outperformed other models with an AUC value of 0.947, and the scorecard model has an AUC of 0.915. Additionally, we have established an online assessment system for early identification of MAS secondary to SLE.

**Conclusion:**

Machine learning models can significantly improve the diagnostic accuracy of MAS secondary to SLE, and the diagnostic scorecard model can facilitate personalized probabilistic predictions of disease occurrence in clinical environments.

**Supplementary Information:**

The online version contains supplementary material available at 10.1186/s13075-024-03330-9.

## Introduction

Systemic Lupus Erythematosus (SLE) is a complex and heterogeneous disease characterized by abnormal activation of the immune system, leading to systemic impairment of multiple organs and tissues [1]. According to the latest data, the incidence rate of SLE in China is approximately 47.15–47.96 per 100,000, and this proportion is gradually increasing [2]. This has imposed a significant burden on individual health and socioeconomic aspects. Macrophage Activation Syndrome (MAS) is a rare, severe, and potentially fatal complication of SLE. Previous studies have reported that approximately 0.9-4.6% of patients with SLE eventually develop into MAS [3]. Patients with MAS secondary to SLE have a rapid disease progression and are prone to developing multi-organ dysfunction within a short period of time. The advanced stage of the disease poses significant challenges to treatment and carries a high risk of mortality, estimated to be around 4–19% [4]. Therefore, early identification of MAS secondary to SLE patients is particularly important to improve the quality of patient survival and optimize survival outcomes. However, It is difficult to identify the MAS secondary to SLE due to the lack of specific symptoms and signs. In addition, MAS sometimes serves as the starting performance in patients with SLE, resulting in difficulty in diagnosis [5].

Currently, research on MAS secondary to SLE is predominantly reported in the form of case studies and small-sample retrospective clinical studies. A retrospective study in Morocco highlighted the need to be vigilant for the development of secondary MAS in SLE patients presenting with unexplained fever, hyperlipidemia, elevated ferritin levels, and high SLE Disease Activity Index (SLEDAI) scores [6]. Another study conducted in China revealed that fever, elevated lactate dehydrogenase levels, splenomegaly, hyperferritinemia, and hypoalbuminemia are the most common clinical characteristics of secondary MAS in SLE [7]. In recent years, both domestic and international research has focused on novel biomarkers associated with the onset of secondary MAS in SLE. A Study found a significant increase in serum CXCL9 levels and sTNFR-II levels in patients with SLE-related MAS compared to SLE alone (*p* < 0.05) [8]. Furthermore, serum sCD163 has been identified as a predictive factor for diagnosing secondary MAS in SLE patients, exhibiting sensitivity and specificity rates of 59% and 86%, respectively [9]. However, these studies do not provide consistent conclusions, and significant diagnostic factors of MAS secondary to SLE remain unclear.

In recent years, the application of machine learning (ML) in autoimmune diseases has developed rapidly. Predictive modeling based on ML has broad prospects for improving disease diagnosis and prognosis [10]. Previously, researchers have employed ML algorithms to filter critical predictive features within SLE datasets, subsequently developing the “SLE Risk Probability Index”, which boasts an accuracy rate of up to 94.8% in the identification of SLE [11]. A study has shown that ML can effectively predict the likelihood of disease recurrence in patients with rheumatoid arthritis by analyzing ultrasound and blood test results at baseline. Notably, the XGBoost algorithm performed the best, with an area under the receiver operating characteristic curve (AUC) of 0.747 [12]. Another study employed unsupervised ML to categorize anti-melanoma differentiation-associated protein 5 (anti-MDA5) positive patients with dermatomyositis into three distinct subtypes and established a decision tree algorithm to forecast their clinical characteristics and prognosis. However, to date, no study has attempted to train ML models using clinical information for the early diagnosis of MAS secondary to SLE [13].

In this study, we employed ML techniques to develop a predictive model for secondary MAS in patients with SLE. By utilizing commonly available clinical data such as physical examination and hematological indices, we identified the predictive factors for MAS secondary to SLE and constructed a diagnostic scoring system. This system enhances the clinical assessment of disease likelihood and facilitates the prompt detection of secondary MAS in patients with SLE.

## Method

### Study population

This study included 188 patients diagnosed with SLE (94 patients) or MAS secondary to SLE (94 patients) between May 2012 and January 2023 at the Union Hospital of Tongji Medical College, Huazhong University of Science and Technology. Furthermore, data from patients with SLE and MAS secondary SLE from the Second Xiangya Hospital of Central South University, the Central Hospital of Wuhan, the Zhongnan Hospital of Wuhan University, and the Second Affiliated Hospital of Zhejiang University School of Medicine were collected as an external validation set in this study. The selection of SLE patients complied with the 1997 ACR classification criteria [14]. The selection of MAS secondary to SLE complied with both the 1997 ACR classification criteria and five of the eight HLH-2004 diagnostic criteria [15]. Exclusion criteria were: (1) < 14 years of age; (2) history of combined tumor and other autoimmune diseases; (3) a large amount of missing data. The study was approved by the Ethics Committee of Union Hospital, Tongji Medical College, Huazhong University of Science and Technology.

### Candidate predictive variables

The clinical information collected in this study encompassed various aspects of patient characteristics, clinical features, and laboratory parameters. The dataset included demographic information such as age and gender. Clinical features consisted of the Systemic Lupus Erythematosus Disease Activity Index (SLEDAI), highest recorded body temperature, duration of fever, reported symptoms, as well as laboratory indicators such as white blood cell count (WBC), hemoglobin (HB), platelet count (PLT), total bilirubin (TBil), serum electrolytes, serum creatinine (SC), alanine aminotransferase (ALT), aspartate aminotransferase (AST), alkaline phosphatase (ALP), total protein (TP), albumin (ALB), globulin (GLB), serum ferritin (SF), triglycerides (TG), serum low-density lipoprotein cholesterol (LDL), serum high-density lipoprotein cholesterol (HDL), lactate dehydrogenase (LDH), percentages of CD3 T cells, CD8 T cells, CD19 T cells, CD4 T cells, NK cells, as well as levels of interleukin-2 (IL-2), interleukin-4 (IL-4), interleukin-6 (IL-6), interleukin-10 (IL-10), tumor necrosis factor-alpha (TNF-α), interferon-gamma (IFN-γ), antinuclear antibody (ANA), anti-double stranded DNA antibodies (Anti-dsDNA), C-reactive protein (CRP), procalcitonin (PCT), erythrocyte sedimentation rate (ESR), D-dimer, activated partial thromboplastin time (APTT), fibrinogen (FIB), thrombin time (TT), prothrombin time (PT), immunoglobulin A (IgA), immunoglobulin G (IgG), immunoglobulin M (IgM), C3, and C4, totaling 91 variables. The data from the external validation set were collected from four other domestic hospitals.

### Data processing and feature engineering

In order to obtain high-quality data, we handled missing values using multiple methods. Variables missing < 5% were populated with plurality and mean values, while variables missing between 5% and 20% were populated using random forests. We performed Mann-Whitney U tests on the data before and after interpolation to ensure that no significant change in data distribution was produced by our interpolation algorithms.

To maximize data retention, we incorporated the clinically accepted normal range as prior knowledge. More specifically, we added some new derived features to indicate whether the value exceeded the upper and lower limits of the empirical range (represented as 1 for values exceeding the upper limit, -1 for values below the lower limit, and 0 for values within the normal range). We identified and processed outliers using box plots. Finally, we normalized the data via Z-scoring to avoid scaling differences across units. Following this procedure, the data conformed to the standard normal distribution.

In the external dataset, patients with missing values were excluded from the evaluation. The remaining samples were normalized using the Z-score model, which had been previously established in the processing of the training dataset.

### Feature selection

High-dimensional data is prone to noise during the modeling process and often requires dimensionality reduction. To address this issue, we conducted a two-stage feature selection process. In the first stage, the Pearson correlation coefficient and Variance Inflation Factor (VIF) were used to analyze the correlation and collinearity among various features. Irrelevant features and some features with multicollinearity with the diagnostic outcomes were removed. Derived features with clinical significance were constructed based on clinical experience. In the second stage, the Least Absolute Shrinkage and Selection Operator (LASSO) with 5-fold cross-validation was used to select the remaining features. The variables selection using LASSO is depicted in Supplemental Figure S1.

### Model and evaluation

**Fig. 1:**
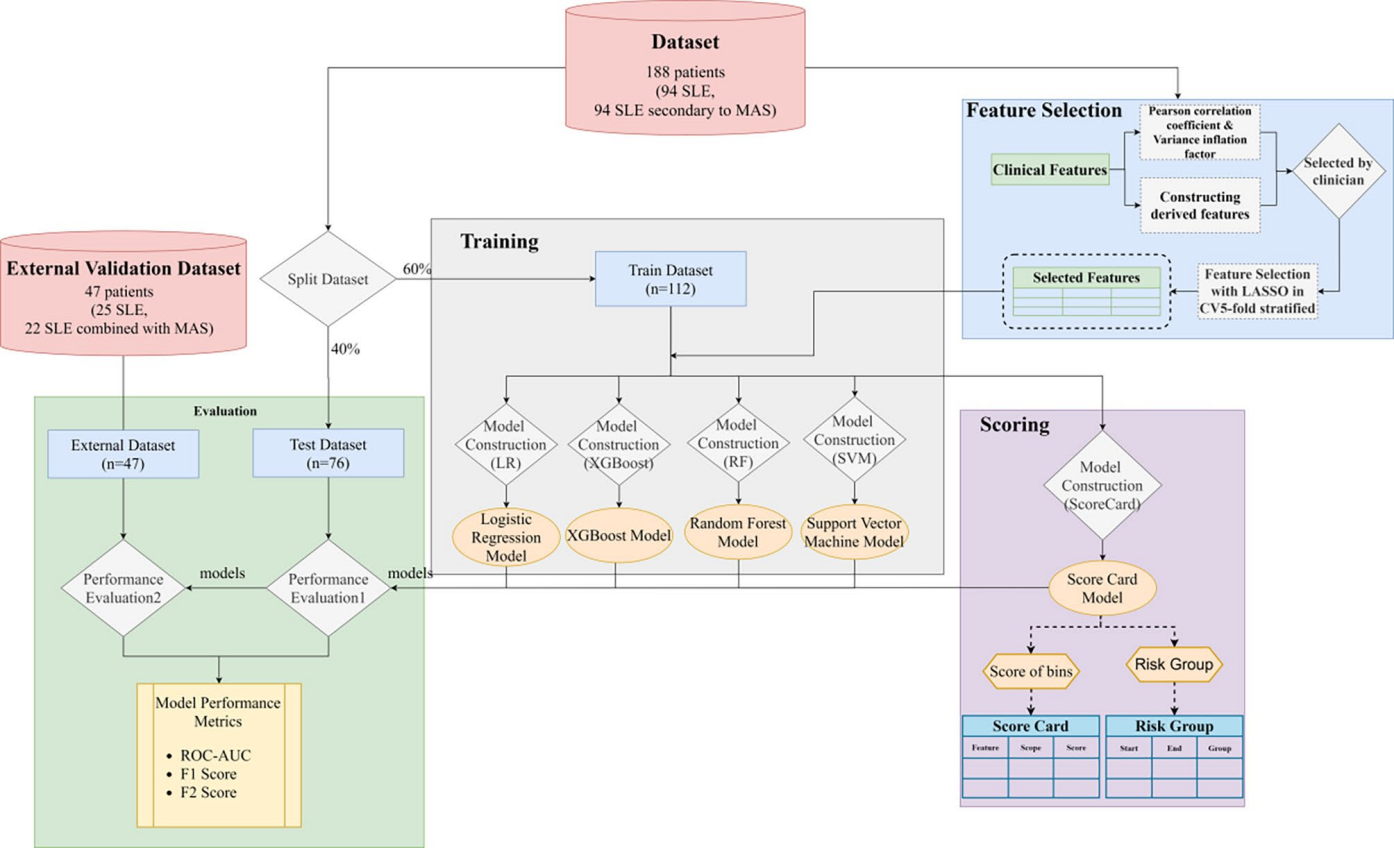
Illustrative overview of the development of diagnostic machine learning model and scoring system for MAS secondary to SLE

The study employed five classification models, Logistic Regression (LR), Extreme Gradient Boosting (XGBoost), Random Forest (RF), Support Vector Machine (SVM), and Scorecard model to construct an evaluation model for MAS secondary to SLE(Fig. 1). Regarding parameter optimization, a L2 regularization penalty was introduced into the logistic regression model to address overfitting resulting from limited data. The L2 regularization term penalizes high complexity weights by adding the sum of the squared weights to the loss function. We used grid search to optimize relevant hyperparameters and utilized the leave-one-out method for model validation (Supplemental Table S1). The leave-one-out method is a type of cross-validation technique where one data point from the training set is used as the validation set, while the rest of the data is used for training. This method is especially reliable for accurately evaluating the model’s performance on the training set when the dataset is small.

For model evaluation, the performances of these models were assessed and compared across the test set and the external dataset. Considering the high risk of mortality in MAS secondary to SLE, and the potential adverse outcomes resulting from misdiagnosis, we believed that the model should assign varying levels of importance to different types of errors. In the diagnosis of high-risk diseases, false negatives (FN) should be minimized as much as possible. Therefore, we introduced F1 Score and F2 Score as evaluation metrics. The F-Score is the harmonic mean of precision and recall (formula 1–1). The F2 Score applies a weighted penalty to FN errors by assigning a higher weight to sensitivity (by setting β = 2 in formula 1–1), which compels the model to better address cases of missing diagnosis.

1-1 $$F - Score = (1 + {\beta ^2}) \cdot \frac{{\Pr ecision\, \cdot Recall}}{{{\beta ^2} \cdot \Pr eicision + \operatorname{Re} call}}$$

### Diagnostic scorecard

In this study, we have developed a diagnostic scoring system to predict the probability of secondary MAS in SLE [16].This system utilizes the same set of features as the diagnostic models.

The algorithm first discretized the value range of features into bins. For categorical features, each category was treated as a separate bin, and for continuous features, the chi-square binning algorithm was employed. The chi-square binning algorithm pre-divides the value range of the continuous feature into 20 bins and performs chi-square tests on adjacent pairs of bins. The pair with a *p*-value less than 0.05 and the highest chi-square value was merged. This process was iterated until no further merging was possible or the minimum bin number was met.

Step 2 involved calculating the Weight of Evidence (WOE) values for each bin. The sample feature values were then transformed into the corresponding bin’s woe values, which were used to train a logistic regression model. Calculate Woe according to the following formula:

2-1 $$Wo{E}_{i}=ln\left(\frac{SLE\%}{MAS\%}\right)$$

Calculate the coefficients A and B for the score transformation function according to the following formulas:

3-1 $$Score=A-B\times ln\left(odds\right)$$

3-2 $$B=\frac{{PD}_{0}}{ln2}$$

3-3 $$A={P}_{0}+B\times ln\left(odds\right)$$

Among them, the odds value represented the ratio of disease to non-disease, P0 was the baseline score at that ratio, PD0 was the score reduction when the ratio doubled, the A and B were coefficients of the scoring function. In this paper, the initial odds value was set to 1/19, P0 was set to 70, PD0 was set to 4.14, and the coefficients A and B were obtained as 52.41 and 5.97, respectively.

To facilitate the assessment of the probability of disease corresponding to the scores, we established the probability thresholds which identified the highest score that satisfied the predefined disease risk (defined as the conditional probability of developing MAS secondary to SLE at a certain score). In practical operations, accurately estimating probabilities through sample frequencies was challenging due to limited sample sizes. Instead, we utilized Gaussian kernel density estimation to estimate probability density functions for positive and negative samples. Gaussian kernel density estimation is a non-parametric method for estimating probability density functions (PDFs). The integration of the PDFs over a unit-sized interval determined the probability of falling within that interval. Considering that positive (MAS secondary to SLE) and negative (SLE) samples were mutually exclusive events, the disease risk of the patient within a determined score interval was equivalent to the probability of a positive sample falling into that interval.

## Results

### Baseline data

In this study, we conducted an analysis of the differences in age at onset, gender, hepatosplenomegaly, neurological symptoms, and mortality rate among 94 patients with SLE and 94 patients with MAS secondary to SLE based on their baseline data. We observed that patients with secondary MAS had an earlier age of onset compared to those with isolated SLE (median age [Q1, Q3]: 44 years [33.2, 52.8] vs. 31.5 years [24.2, 46.8] respectively, *p* < 0.001). However, there was no significant difference in gender distribution between the SLE group and the MAS secondary to SLE group (males/females: 8.51%/91.5% vs. 16.0%/84.0%, *p* = 0.182). The incidence of hepatomegaly was significantly higher in the MAS secondary to SLE group compared to the SLE group (9.57% vs. 1.06%, *p* = 0.023). Similarly, the incidence of splenomegaly was also higher in the MAS secondary to SLE group compared to the SLE group (38.3% vs. 12.8%, *p* < 0.001). Patients with MAS secondary to SLE had a higher proportion of neurological and psychiatric system involvement compared to SLE patients (2.13% vs. 16%, *p* = 0.02). Furthermore, there was a significant increase in the mortality rate in the MAS secondary to SLE group compared to the SLE group (0/0% vs. 15/16%, *p* < 0.001)(Supplemental Table S2). The baseline of external validation can be found in Supplemental Table S3 and Supplemental Table S4.

### Identification of diagnostic factors for MAS secondary to SLE

A screening of 149 features constructed from 91 clinical indicators was conducted to identify stable and representative diagnostic factors(Supplemental Figure S2 and Supplemental Table S5). Two composite features (Pancytopenia and Liver Damage) were used to replace their corresponding original indicators (HB, PLT, WBC, ALT, AST). The LASSO combined with 5-fold cross-validation identified 13 significant features from the candidate variables, as follows: maximum temperature, duration of fever, serum sodium, TG, HDL, LDH, ferritin, CRP, FIB, PT, TNF-α, pancytopenia, and liver damage.

### Diagnostic models

**Fig. 2:**
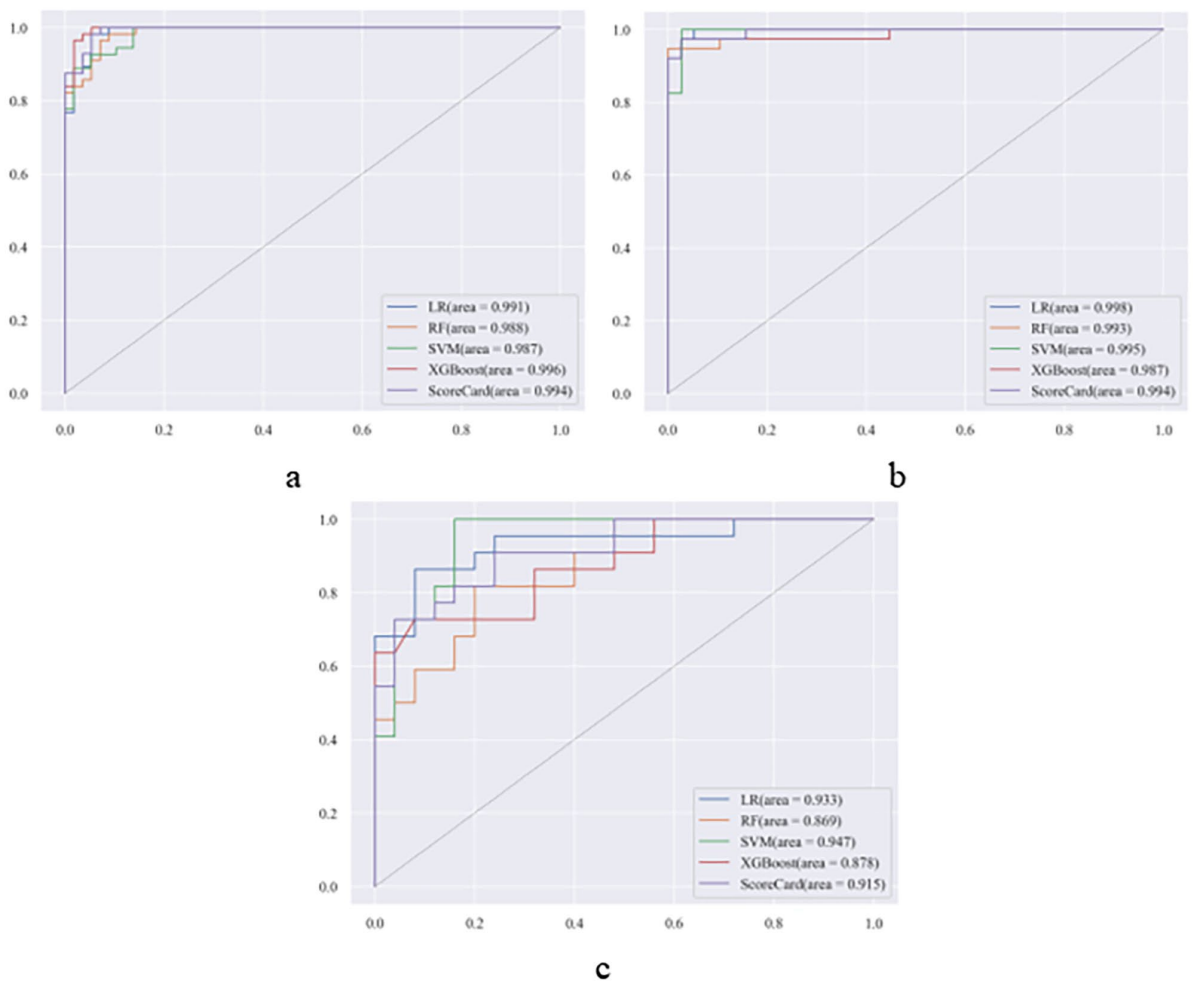
ROC curves of the Machine Learning Model for Early Identification of MAS secondary to SLE on Internal Data Set and External Validation

The performance of the models was assessed utilizing the AUC, F1 score, and F2 score (Table 1) across the validation dataset, test dataset, and external dataset. All algorithms performed well across the three datasets. LR yielded an AUC of 0.998 on the test dataset vs. 0.933 on the external dataset and an F2 score of 0.952 on the test dataset vs. 0.885 on the external dataset. SVM achieved an AUC of 0.995 on the test dataset vs. 0.947 on the external dataset and an F2 score of 0.995 on the test dataset vs. 0.893 on the external dataset. The score card achieved an AUC of 0.994 on the test dataset vs. 0.915 on the external dataset and an F2 score of 0.936 on the test dataset vs. 0.721 on the external dataset(Fig. 2). The LR model exhibited superior performance on the test dataset and the SVM performed best on the external dataset.

**Table 1:** Model Performance Evaluation on Internal Datasets and External Validation Datasets (ROC-AUC, F1 Score, F2 Score)

	Validation			Test			External Validation		
	AUC	F1	F2	AUC	F1	F2	AUC	F1	F2
LR	0.991	0.937	0.932	0.998	0.960	0.952	0.933	0.851	0.885
RF	0.988	0.948	0.968	0.993	0.960	0.952	0.869	0.720	0.776
SVM	0.987	0.926	0.926	0.995	0.988	0.995	0.947	0.870	0.893
XGBoost	0.996	0.973	0.979	0.987	0.946	0.931	0.878	0.741	0.833
ScoreCard	0.994	0.957	0.972	0.994	0.959	0.936	0.915	0.789	0.721

**Fig. 3:**
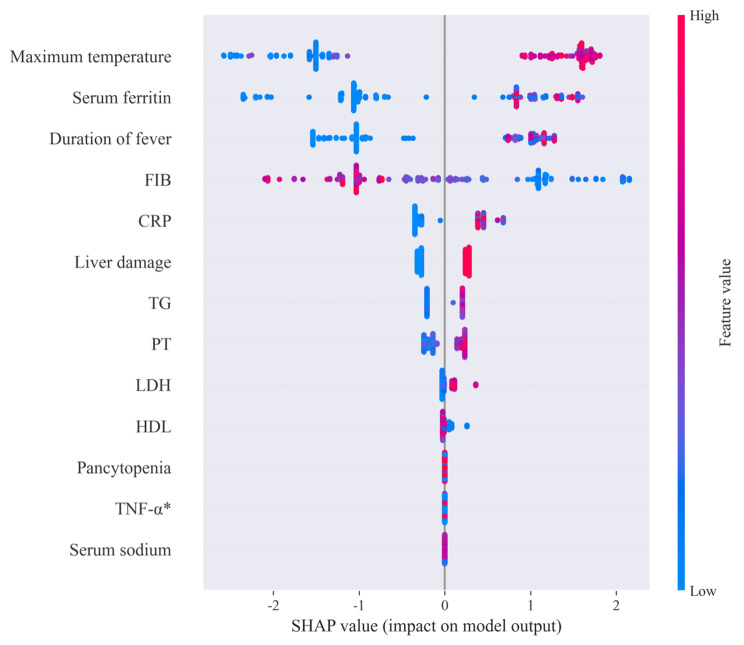
SHAP analysis plot of the early identification model for MAS secondary to SLE

Figure 3 illustrates the analysis plot for all samples, showcasing the 13 distinct features. The plot reveals that both fever temperature and duration are significant contributors to the prediction of MAS secondary to SLE. When the fever temperature is high and the duration is prolonged, the probability of developing the disease is relatively higher. Additionally, elevated ferritin levels have a notable impact on the prediction outcome. As ferritin levels exceed the average, the probability of developing MAS secondary to SLE increases with the increasing values. Furthermore, when FIB levels are above the average, the relative probability of developing the disease is lower. However, when FIB is below the average, the impact of this indicator on the probability of MAS secondary to SLE significantly increases as the values decrease. In summary, our diagnostic models exhibited robust performance across all three datasets, with the LR and the SVM model yielding the most favorable outcomes. Through the SHAP analysis plot, we identified maximum temperature, serum ferritin, duration of fever, and FIB as the most influential variables in our models.

### Diagnostic scorecard

**Fig. 4:**
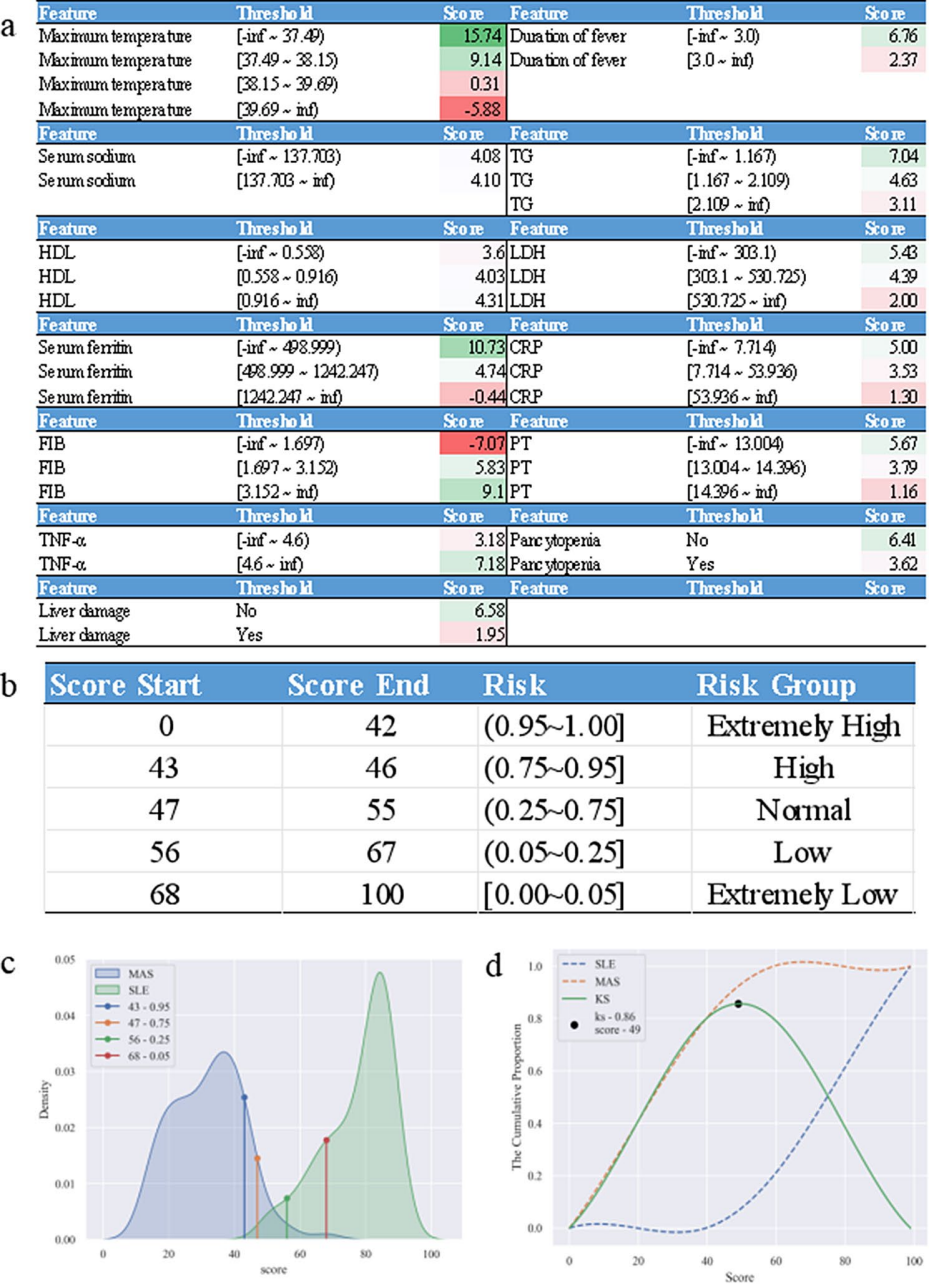
Score card for diagnostic assessment of MAS secondary to SLE, Diagnostic Threshold Grouping, and Score Distribution. **a**. score card for diagnostic assessment of MAS secondary to SLE. **b** Diagnostic grouping and threshold scores. **c** Probability Density Plots of Scores for SLE Patients and MAS secondary to SLE Patients. **d** KS (Kolmogorov-Smirnov) curve for the score card

To enhance the convenience of clinical utilization, we developed a scoring system (Fig. 4a). The score card system achieved AUC of 0.994 on the test set and an AUC of 0.915 on the external dataset(Table 1). The comparable AUC values indicate that the performance of this system was similar to the diagnostic model (LR), suggesting that binning did not significantly alter the distribution of the data, thus confirming the rationality of the binning algorithm.

The Kolmogorov-Smirnov (KS) curve was utilized to analyze the score distribution(Fig. 4d). The KS curve showcased the cumulative distribution of the patient population of MAS secondary to SLE (the orange curve) alongside the patient population of SLE (the blue curve) as the score increased from 0 to 100. Additionally, it displayed the difference between the two curves (the green curve). It was evident that the MAS secondary to SLE population accumulated rapidly at lower scores, whereas the SLE population tends to gather more swiftly at higher scores, indicating that the MAS secondary to SLE population primarily occupied the low score range, whereas the SLE population was mainly concentrated in the high score range. The highest point on the green line (score = 49) served as an effective differentiator between the SLE and MAS secondary to SLE populations. Hence, we have chosen this specific score as the threshold for the scorecard.

The density plot (Fig. 4c) showed that the scores of MAS secondary to SLE patients (blue line) were mainly distributed in the low score range, which was consistent with the KS curve. By establishing probability thresholds for developing MAS secondary to SLE at 95%, 75%, 25%, and 5%, we delineated the scores corresponding to the five diagnostic groups depicted in Fig. 4b: extremely low (below 5%, 68∼100), low (5–25%, 56∼67), normal (25–75%, 47∼55), high (75–95%, 43∼46), and extremely high (above 95%, 0∼42).

We have developed an online MAS secondary to SLE scoring tool, which could be accessed through an online Webpage [17]. This tool facilitates both physicians and patients in the accurate diagnosis of MAS secondary to SLE based on the indicators submitted online. The indicators that fall within a threatening range will be highlighted, serving as a helpful reminder. In conclusion, our diagnostic scorecard exhibited comparable performance to our diagnostic models across all three datasets. Furthermore, we observed significant differences in the distribution of scores between the two patient groups. And we determined the bordering scores of the five diagnostic groups we defined.

## Discussion

The early diagnosis of MAS secondary to SLE remains challenging. The typical symptoms of MAS, such as fever and hematological abnormalities, may occur in SLE patients due to disease activity, making early diagnosis extremely difficult. At present, the diagnostic criteria for MAS still follow the HLH-2004 standards. Previous studies have shown that the critical values of the diagnostic indicators for MAS may be influenced by different autoimmune diseases [18]. Therefore, it is necessary to conduct personalized research on the clinical predictive features of MAS secondary to SLE. To the best of our knowledge, this is the first study to focus on the prediction of patients with MAS secondary to SLE.

We employed an ML algorithm to construct an early detection model for MAS secondary to SLE. Compared to traditional statistical methods, ML demonstrates superior predictive accuracy in complex data scenarios [19]. The LR model we employed, incorporating L2 regularization, effectively mitigates overfitting in scenarios with limited samples in rare diseases. This approach enhances the efficiency of the model in disease recognition. Additionally, the XGBoost model and SHAP analysis method we utilized provide feature importance assessments, aiding in the understanding of the predictive process of the model. In our study, we introduce for the first time a novel diagnostic scoring system designed to assess the probability of developing MAS secondary to SLE. This scoring system is capable of automatically calculating a total score and evaluating the likelihood of disease based on 13 common indicators entered by clinical physicians. The scoring system quantifies the impact of each predictive variable on the probability of disease occurrence by establishing specific numerical ranges and assigning unique score values. Physicians can utilize these scored values to intuitively grasp the patient’s performance level in relation to a particular predictive feature, which aids in more accurate and detailed clinical assessments. Furthermore, the selected predictive variables are readily obtainable in clinical practice and involve low testing costs. When integrated with an online scoring system, they can promptly identify patients in the early stages of MAS secondary to SLE. The performance and clinical utility of the model have also been validated using data from multiple centers within the country. The external validation has shown excellent performance, indicating that the model can be effectively applied in clinical practice.

Fever presents as an exceedingly common symptom among patients with MAS secondary to SLE, with a significant number experiencing prolonged episodes of fever before receiving a definitive diagnosis. It may be attributed to the inflammatory cytokine storm associated with MAS, lupus flare, or concurrent infections. The persistence of fever serves as a crucial indicator warranting heightened vigilance for the potential development of secondary HLH. In our study, nearly all patients exhibited hyperferritinemia during the progression of the disease. The HLH-2004 criteria include a ferritin level > 500 µg/mL as one of the diagnostic criteria. However, it is recognized that severe infections, malignancies, and other conditions can also cause ferritin levels to rise to 500 µg/L or above, leading to a growing number of scholars advocating for a reassessment of the ferritin threshold in HLH diagnosis [20]. In a retrospective study focusing on adult HLH, the average ferritin level was 70,398 ng/mL, and the optimal cutoff value for ferritin was proposed to be 16,000 ng/mL (sensitivity 79.4%, specificity 79.2%) [21]. Lehmberg et al. compared 123 HLH patients with 320 patients with other causes of hyperferritinemia, suggesting a threshold value of 2,000 µg/L for ferritin (sensitivity 70%, specificity 68%) [22]. Our study further supports the view that ferritin levels above 1,242.25 µg/mL possess higher predictive value for secondary MAS associated with SLE. Our study has identified for the first time that low levels of high-density lipoprotein are predictive factors for MAS secondary to SLE. Previous studies have shown that low serum HDL levels are independent risk factors influencing the prognosis of secondary HLH [23], and the occurrence of low HDL levels is higher in hemophagocytic syndrome patients compared to hypertriglyceridemia [24]. Currently, the HLH-2004 diagnostic criteria only mention hypertriglyceridemia (TG > 3 mmol/L), and we suggest that more attention should be paid to abnormal changes in high-density lipoprotein levels in patients with MAS secondary to SLE. Previous studies have shown that compared to HLH patients, MAS patients have higher levels of CRP, and a cutoff value of 90 mg/L has been identified to differentiate between these two conditions (AUC: 0.87, sensitivity: 0.85, specificity: 0.83) [25]. In our study, patients with MAS secondary to SLE had higher levels of CRP compared to those without MAS. Consistently, similar results were reported by Ahn et al [26]. High levels of CRP may be associated with the activity of the primary disease and secondary infections in MAS. Monitoring the dynamic changes in CRP levels can help differentiate between MAS and disease activity [27]. According to the study conducted by Kostik et al., hyponatremia can serve as a biomarker for early recognition of MAS [28]. The occurrence of hyponatremia may be related to the impact of MAS on renal tubular function [29]. Our research indicates that hyponatremia shows promise as a distinguishing factor between SLE and MAS secondary to SLE. However, its discriminative power is not as robust as that of other indicators.The pathogenesis of MAS involves a variety of cytokine storms and excessive inflammatory responses [30]. Currently, the cytokine profile and underlying mechanisms associated with MAS secondary to SLE remain unclear. Some studies have indicated elevated levels of TNF-α-related molecules in MAS secondary to SLE, which can serve as diagnostic markers for the transition from active SLE to MAS [31]. During the remission phase of MAS, multiple cytokines including TNF-α exhibit decreased levels compared to the active phase [32]. However, lower levels of TNF-α were found in SLE patients with MAS compared to single SLE patients in the present study. Different results may be attributed to confounding factors, such as the time of TNF-α measurements. Because our cytokine measurements were only performed at admission, we need to interpret these findings with caution. In the future, through dynamic monitoring of changes in TNF-α levels throughout the course of MAS, we can better understand the role of TNF-α in MAS secondary SLE.

Certainly, this study presents several limitations. The small sample size used may limit the generalizability of the findings and compromise the diagnostic accuracy due to potential data imbalance. Additionally, Although the risk of overfitting was reduced in this study through techniques such as cross-validation, there is still a possibility of overfitting when using complex models and a large number of features in small-sample situations. Moreover, the limited availability and potential missing data of certain examination indicators at our center may result in overlooked or underestimated clinical information related to the disease. In the future, it is recommended to establish a multi-center prospective study specifically targeting MAS secondary to SLE in order to further confirm the efficacy of the model.

## Conclusion

In conclusion, we have developed a predictive model and scoring card based on clinical features to predict MAS secondary to SLE, which can assist clinicians in implementing better-personalized disease management, facilitating dynamic monitoring and early warning of the disease progression.

### Electronic supplementary material

Below is the link to the electronic supplementary material.

Supplementary Material 1. Supplement Figure S1 Variables selection using the LASSO regression analysis with 5-fold cross-validation. a coefficient profile against the log (lambda). b parameter selection of deviance in the LASSO regression, log of the best lambda was – 1.5 in this study shown as red line. Supplement Table. S1 Model hyperparameters. Supplement Table S2 Baseline demographic and clinical characteristics of SLE patients and MAS secondary to SLE. Supplement Table S3 Baseline demographic and clinical characteristics of SLE patients and MAS secondary to SLE for external validation. Supplement Table S4 baseline demographic and clinical characteristics of SLE patients and MAS secondary to SLE for original set and validation set. Supplement Figure S. 2 Pearson correlation coefficients of clinical features. The features marked with an asterisk (*) represent derived features calculated from the original feature and its corresponding normal range. Supplement Table S5 Variance inflation factors of clinical features. The features marked with an asterisk (*) represent derived features calculated from the original feature and its corresponding normal range.

## Data Availability

No datasets were generated or analysed during the current study.

## Abbreviations

AUC-ROC: Area Under the Receiver Operating Characteristic Curve
FN: False Negative
LASSO: The Least Absolute Shrinkage and Selection Operator
LR: Logistic regression
MAS: Macrophage Activation Syndrome
ML: Machine learning
PDFs: Probability density functions
RF: Random Forest
SLE: Systemic lupus erythematosus
SVM: Support Vector Machine
VIF: Variance inflation factor
WOE: Weight of Evidence
XGBoost: Extreme Gradient Boosting
