# Supplementary material for: Early identification of macrophage activation syndrome secondary to systemic lupus erythematosus with machine learning
Source: Arthritis Res Ther. 2024 May 9;26:92. doi: 10.1186/s13075-024-03330-9 (PMC11080238; doi:10.1186/s13075-024-03330-9)
Supplement: Supplementary file 1 — Supplementary Material 1. Supplement Figure S1 Variables selection using the LASSO regression analysis with 5-fold cross-validation. a coefficient profile against the log (lambda). b parameter selection of deviance in the LASSO regression, log of the best lambda was – 1.5 in this study shown as red line. Supplement Table. S1 Model hyperparameters. Supplement Table S2 Baseline demographic and clinical characteristics of SLE patients and MAS secondary to SLE. Supplement Table S3 Baseline demographic and clinical characteristics of SLE patients and MAS secondary to SLE for external validation. Supplement Table S4 baseline demographic and clinical characteristics of SLE patients and MAS secondary to SLE for original set and validation set. Supplement Figure S. 2 Pearson correlation coefficients of clinical features. The features marked with an asterisk (*) represent derived features calculated from the original feature and its corresponding normal range. Supplement Table S5 Variance inflation factors of clinical features. The features marked with an asterisk (*) represent derived features calculated from the original feature and its corresponding normal range. [file 13075_2024_3330_MOESM1_ESM.docx]

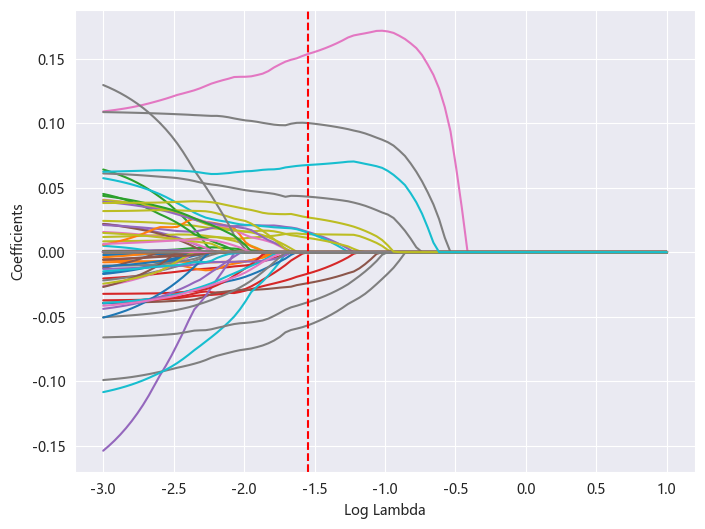

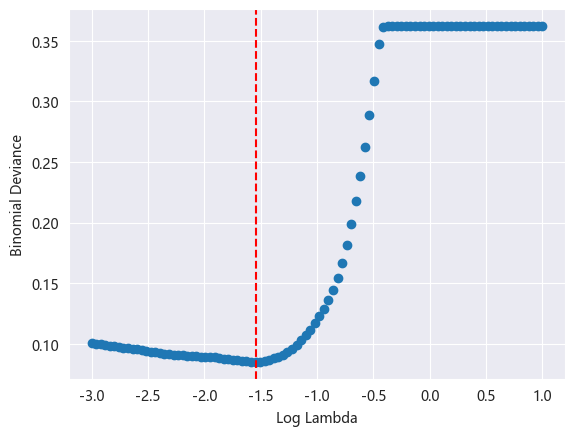


a

b

Supplement Figure S1 Variables selection using the LASSO regression analysis with 5-fold cross-validation. a coefficient profile against the log (lambda). b parameter selection of deviance in the LASSO regression, log of the best lambda was -1.5 in this study shown as red line.

Supplement Table S1 Model hyperparameters.

| Model | LR | RF | Xgboost | SVM | ScoreCard |
| --- | --- | --- | --- | --- | --- |
| hyperparameters | C: 0.1  class_weight: None  max_iter: 1000  penalty: l2 | max_depth: 2  max_features: sqrt  min_samples_leaf: 2  min_samples_split: 2  n_estimators: 50 | max_depth: 3  booster: gbtree  base_score: 0.5  n_estimators: 100  colsample_bytree: 0.8  max_delta_step: 0.1  eval_metric: auc | C: 1  degree: 3  gamma: scale  kernel: rbf | C: 0.1  class_weight: None  max_iter: 100  penalty: l2 |

Supplement Table S2 Baseline demographic and clinical characteristics of SLE patients and MAS secondary to SLE.

| **Variable** | **SLE Group*（*N=94）** | **MAS secondary to SLE Group*（*N=94）** | **p.overall** |
| --- | --- | --- | --- |
| Age(years) | 44.0 [33.2;52.8] | 31.5 [24.2;46.8] | <0.001 |
| Sex, n(%) |  |  | 0.182 |
| Female | 86 (91.5%) | 79 (84.0%) |  |
| Male | 8 (8.51%) | 15 (16.0%) |  |
| BMI (kg/m²) | 21.8 [19.5;24.5] | 19.1 [18.2;21.4] | <0.001 |
| SLEDAI | 9.50 [6.25;14.0] | 14.5 [11.0;17.0] | <0.001 |
| Pulmonary infection, n(%) |  |  | <0.001 |
| No | 48 (51.1%) | 18 (19.1%) |  |
| Yes | 46 (48.9%) | 76 (80.9%) |  |
| Immunodepressant, n(%) |  |  | <0.001 |
| No | 34 (36.2%) | 72 (76.6%) |  |
| Yes | 60 (63.8%) | 22 (23.4%) |  |
| Pathogenic detection, n(%) |  |  | <0.001 |
| No | 78 (83.0%) | 47 (50.0%) |  |
| Yes | 16 (17.0%) | 47 (50.0%) |  |
| Maximum temperature(℃) | 36.9 [36.8;37.5] | 39.5 [39.0;40.0] | <0.001 |
| Duration of fever(d) | 0.00 [0.00;1.00] | 19.5 [11.5;30.0] | <0.001 |
| Rash: |  |  | 0.136 |
| No | 62 (66.0%) | 51 (54.3%) |  |
| Yes | 32 (34.0%) | 43 (45.7%) |  |
| Joint pain, n(%) |  |  | 1.000 |
| No | 65 (69.1%) | 64 (68.1%) |  |
| Yes | 29 (30.9%) | 30 (31.9%) |  |
| Hepatomegaly, n(%) |  |  | 0.023 |
| No | 93 (98.9%) | 85 (90.4%) |  |
| Yes | 1 (1.06%) | 9 (9.57%) |  |
| Splenomegaly, n(%) |  |  | <0.001 |
| No | 82 (87.2%) | 58 (61.7%) |  |
| Yes | 12 (12.8%) | 36 (38.3%) |  |
| Enlarged lymph node, n(%) |  |  | <0.001 |
| No | 72 (76.6%) | 29 (30.9%) |  |
| Yes | 22 (23.4%) | 65 (69.1%) |  |
| Neuropsychiatric symptoms, n(%) |  |  | 0.002 |
| No | 92 (97.9%) | 79 (84.0%) |  |
| Yes | 2 (2.13%) | 15 (16.0%) |  |
| WBC at admission (×10^9^/L) | 5.36 [3.56;7.77] | 3.99 [2.39;6.82] | 0.003 |
| WBC (×10^9^/L) | 4.79 [2.95;6.44] | 2.16 [1.61;3.47] | <0.001 |
| Days to minimum WBC (d) | 0.00 [0.00;4.00] | 3.50 [1.25;9.75] | <0.001 |
| HB at admission (g/L) | 101 (20.5) | 93.5 (17.8) | 0.011 |
| HB (g/L) | 98.50 [81.2;110] | 75.5p [64.2;89.0] | <0.001 |
| Days to minimum HB (d) | 4.00 [0.00;5.75] | 6.00 [3.00;15.0] | <0.001 |
| PLT at admission (×10^9^/L) | 173 [124;222] | 110 [64.2;172] | <0.001 |
| PLT (×10^9^/L) | 156 [105;210] | 52.0 [30.8;96.0] | <0.001 |
| Days to minimum PLT (d) | 0.50 [0.00;5.00] | 7.00 [3.00;14.8] | <0.001 |
| Neu at admission (×10^9^/L) | 3.82 [2.41;6.23] | 2.98 [1.54;5.40] | 0.038 |
| Neu (×10^9^/L) | 3.09 [1.89;4.68] | 1.40 [0.93;2.41] | <0.001 |
| Days to minimum Neu (d) | 0.00 [0.00;4.00] | 5.00 [1.25;13.0] | <0.001 |
| TBIL (umol/L) | 7.85 [6.05;10.3] | 8.90 [5.95;13.5] | 0.136 |
| Serum sodium(mmol/L) | 140 [138;142] | 137 [133;139] | <0.001 |
| Serum potassium(mmol/L) | 3.79 [3.57;4.00] | 3.88 [3.51;4.12] | 0.566 |
| Serum chloride(mmol/L) | 104 (3.61) | 102 (5.19) | 0.006 |
| Serum calcium(mmol/L) | 2.10 (0.15) | 1.98 (0.18) | <0.001 |
| Serum phosphorus(mmol/L) | 1.11 [0.95;1.30] | 1.00 [0.81;1.13] | <0.001 |
| Serum magnesium(mmol/L) | 0.80 (0.10) | 0.82 (0.13) | 0.382 |
| ALP (U/L) | 56.0 [44.5;73.5] | 80.5 [55.0;127] | <0.001 |
| ALT (U/L) | 18.5 [13.0;31.0] | 59.5 [35.0;128] | <0.001 |
| AST (U/L) | 21.5 [17.0;29.0] | 68.0 [41.0;138] | <0.001 |
| Total protein (g/L) | 60.5 (9.65) | 58.7 (10.3) | 0.202 |
| Albumin (g/L) | 32.8 (5.68) | 27.5 (5.11) | <0.001 |
| Globulin (g/L) | 26.7 [22.3;32.8] | 30.4 [25.3;36.3] | 0.003 |
| Serum creatinine (umol/L) | 58.1 [50.6;69.7] | 51.2 [40.6;66.1] | 0.001 |
| TG (mmol/L) | 1.58 [1.12;2.40] | 3.09 [2.18;4.55] | <0.001 |
| HDL (mmol/L) | 1.08 [0.88;1.52] | 0.69 [0.47;1.00] | <0.001 |
| LDL (mmol/L) | 2.37 [2.05;2.92] | 1.88 [1.38;2.71] | <0.001 |
| LDH (U/L) | 245 [212;314] | 635 [373;867] | <0.001 |
| Serum ferritin (μg/L) | 284 [112;583] | 4040 [1723;12204] | <0.001 |
| ESR (mm/h) | 24.5 [10.0;51.8] | 59.5 [30.2;90.8] | <0.001 |
| CRP (mg/L) | 3.41 [2.98;8.75] | 17.9 [6.98;53.0] | <0.001 |
| PCT (ng/mL) | 0.05 [0.05;0.17] | 0.51 [0.13;1.29] | <0.001 |
| CD3+T(%) | 81.0 [70.5;88.6] | 78.0 [71.6;86.0] | 0.299 |
| CD4+T(%) | 34.7 (10.7) | 30.6 (10.5) | 0.008 |
| CD8+T(%) | 38.5 (12.9) | 42.4 (13.5) | 0.049 |
| CD19+B(%) | 11.2 [2.35;19.6] | 11.1 [8.02;20.0] | 0.295 |
| CD3-CD16/56+(NK)(%) | 4.88 [2.38;8.52] | 5.25 [2.29;10.7] | 0.589 |
| D-Dimer (mg/L FEU) | 0.76 [0.34;1.81] | 2.96 [1.46;6.78] | <0.001 |
| FIB (g/L) | 3.09 [2.52;3.86] | 1.58 [0.99;2.44] | <0.001 |
| APTT (s) | 33.7 [31.7;36.5] | 37.9 [32.3;44.6] | <0.001 |
| PT (s) | 12.9 [12.4;13.3] | 13.8 [13.1;15.3] | <0.001 |
| TT (s) | 19.0 [17.5;19.8] | 20.8 [18.4;23.0] | <0.001 |
| Anti-dsDNA positive, n(%) |  |  | 0.233 |
| No | 52 (55.3%) | 61 (64.9%) |  |
| Yes | 42 (44.7%) | 33 (35.1%) |  |
| IL-2 (pg/mL) | 1.71 [1.36;2.24] | 1.96 [1.33;2.64] | 0.137 |
| IL-4 (pg/mL) | 1.62 [1.21;2.04] | 1.86 [1.35;2.76] | 0.007 |
| IL-6 (pg/mL) | 6.36 [3.74;18.2] | 12.5 [5.19;24.3] | 0.018 |
| IL-10 (pg/mL) | 4.19 [2.70;5.98] | 6.15 [4.53;9.04] | <0.001 |
| TNF-α (pg/mL) | 4.10 [1.66;14.6] | 2.19 [1.69;4.66] | 0.012 |
| INF-γ (pg/mL) | 1.88 [1.50;2.42] | 2.65 [1.65;5.74] | <0.001 |
| Serum IgG (g/L) | 13.6 [9.51;18.8] | 16.5 [12.1;21.1] | 0.018 |
| Serum IgA (g/L) | 2.41 [1.46;3.36] | 2.41 [1.44;3.42] | 0.868 |
| Serum IgM (g/L) | 0.84 [0.57;1.30] | 0.98 [0.70;1.39] | 0.055 |
| C3 (g/L) | 0.56 [0.44;0.71] | 0.45 [0.29;0.64] | 0.001 |
| C4 (g/L) | 0.12 [0.10;0.16] | 0.10 [0.06;0.15] | 0.047 |
| EBV-positive, n(%) |  |  | 0.042 |
| No | 23 (24.5%) | 37 (39.4%) |  |
| Yes | 71 (75.5%) | 57 (60.6%) |  |
| Coxsackie virus B positive, n(%) |  |  | 0.863 |
| No | 71 (75.5%) | 73 (77.7%) |  |
| Yes | 23 (24.5%) | 21 (22.3%) |  |
| Enterovirus-positive, n(%) |  |  | 0.101 |
| No | 74 (78.7%) | 63 (67.0%) |  |
| Yes | 20 (21.3%) | 31 (33.0%) |  |
| CMV-positive, n(%) |  |  | 0.204 |
| No | 71 (75.5%) | 79 (84.0%) |  |
| Yes | 23 (24.5%) | 15 (16.0%) |  |
| Hemophagocytosis, n(%) |  |  | <0.001 |
| No | 94 (100%) | 71 (75.5%) |  |
| 1 | 0 (0.00%) | 23 (24.5%) |  |
| Pericardial effusion, n(%) |  |  | <0.001 |
| No | 55 (58.5%) | 29 (30.9%) |  |
| Yes | 39 (41.5%) | 65 (69.1%) |  |
| Pericardial effusion, n(%) |  |  | <0.001 |
| No | 68 (72.3%) | 34 (36.2%) |  |
| Yes | 26 (27.7%) | 60 (63.8%) |  |
| ANA positive titers, n(%) |  |  | 0.724 |
| 1:＜100 | 2 (2.13%) | 2 (2.13%) |  |
| 1:100 | 8 (8.51%) | 5 (5.32%) |  |
| 1:1000 | 25 (26.6%) | 21 (22.3%) |  |
| 1:320 | 10 (10.6%) | 8 (8.51%) |  |
| 1:3200 | 49 (52.1%) | 58 (61.7%) |  |
| Discharge status, n(%) |  |  | <0.001 |
| No | 94(100%) | 79(84%) |  |
| Yes | 0(0%) | 15(16%) |  |

Supplement Table S3 Baseline demographic and clinical characteristics of SLE patients and MAS secondary to SLE for external validation.

|  | **SLE Group*（*N=25）** | **MAS secondary to SLE Group*（*N=22）** | **p.overall** |
| --- | --- | --- | --- |
| Age (years) | 44.2 (14.7) | 37.2 (18.2) | 0.160 |
| Sex, n(%) |  |  | 1.000 |
| Female | 23 (92.0%) | 21 (95.5%) |  |
| Male | 2 (8.00%) | 1 (4.55%) |  |
| BMI (kg/m²) | 22.4 (3.68) | 20.7 (2.38) | 0.075 |
| Maximum temperature(℃) | 37.4 (1.11) | 38.9 (1.17) | <0.001 |
| Duration of fever(d) | 8.32 (18.6) | 18.7 (38.9) | 0.262 |
| Rash: |  |  | 0.270 |
| No | 22 (88.0%) | 16 (72.7%) |  |
| Yes | 3 (12.0%) | 6 (27.3%) |  |
| Joint pain, n(%) |  |  | 0.247 |
| No | 13 (52.0%) | 16 (72.7%) |  |
| Yes | 12 (48.0%) | 6 (27.3%) |  |
| Hepatomegaly, n(%) |  |  | 0.468 |
| No | 25 (100%) | 21 (95.5%) |  |
| Yes | 0 (0.00%) | 1 (4.55%) |  |
| Splenomegaly, n(%) |  |  | 0.063 |
| No | 23 (92.0%) | 15 (68.2%) |  |
| Yes | 2 (8.00%) | 7 (31.8%) |  |
| Enlarged lymph node, n(%) |  |  | 0.995 |
| No | 17 (68.0%) | 14 (63.6%) |  |
| Yes | 8 (32.0%) | 8 (36.4%) |  |
| Neuropsychiatric symptoms, n(%) |  |  | 0.007 |
| No | 25 (100%) | 16 (72.7%) |  |
| Yes | 0 (0.00%) | 6 (27.3%) |  |
| WBC (×10^9^/L) | 5.92 (3.68) | 3.09 (2.76) | 0.004 |
| HB (g/L) | 109 (20.4) | 73.2 (19.4) | <0.001 |
| PLT (×10^9^/L) | 176 (73.5) | 72.2 (71.7) | <0.001 |
| Serum sodium (mmol/L) | 141 (3.33) | 136 (4.09) | <0.001 |
| ALT (U/L) | 22.0 (11.8) | 84.9 (95.4) | 0.006 |
| AST (U/L) | 25.6 (13.6) | 82.2 (69.7) | 0.001 |
| TG (mmol/L) | 1.77 (0.86) | 2.40 (1.12) | 0.037 |
| HDL (mmol/L) | 1.20 (0.39) | 0.85 (0.28) | 0.001 |
| LDH (U/L) | 310 (132) | 532 (351) | 0.010 |
| Serum ferritin (μg/L) | 2699 (2403) | 7155 (13667) | 0.145 |
| CRP (mg/L) | 10.8 (16.0) | 35.6 (41.7) | 0.014 |
| FIB (g/L) | 64.0 (99.3) | 25.2 (89.4) | 0.166 |
| PT (s) | 6.52 (4.81) | 12.7 (1.44) | <0.001 |
| TNF-α (pg/mL) | 4.13 (3.77) | 11.1 (22.1) | 0.155 |

Supplement Table S4 Baseline demographic and clinical characteristics of SLE patients and MAS secondary to SLE for Original set and Validation set.

|  | **Original set (N=188)** | **Validation set (N=47)** | **p.overall** |
| --- | --- | --- | --- |
| Age (years) | 39.1 (14.2) | 40.9 (16.6) | 0.489 |
| Sex, n(%) |  |  | 0.377 |
| Female | 165 (87.8%) | 44 (93.6%) |  |
| Male | 23 (12.2%) | 3 (6.38%) |  |
| BMI (kg/m²) | 21.1 (3.34) | 21.6 (3.21) | 0.313 |
| Maximum temperature(℃) | 38.4 (1.41) | 38.1 (1.38) | 0.200 |
| Duration of fever(d) | 12.6 (17.2) | 13.2 (30.0) | 0.895 |
| Rash: |  |  | 0.013 |
| No | 113 (60.1%) | 38 (80.9%) |  |
| Yes | 75 (39.9%) | 9 (19.1%) |  |
| Joint pain, n(%) |  |  | 0.466 |
| No | 129 (68.6%) | 29 (61.7%) |  |
| Yes | 59 (31.4%) | 18 (38.3%) |  |
| Hepatomegaly, n(%) |  |  | 0.698 |
| No | 178 (94.7%) | 46 (97.9%) |  |
| Yes | 10 (5.32%) | 1 (2.13%) |  |
| Splenomegaly, n(%) |  |  | 0.470 |
| No | 140 (74.5%) | 38 (80.9%) |  |
| Yes | 48 (25.5%) | 9 (19.1%) |  |
| Enlarged lymph node, n(%) |  |  | 0.178 |
| No | 101 (53.7%) | 31 (66.0%) |  |
| Yes | 87 (46.3%) | 16 (34.0%) |  |
| Neuropsychiatric symptoms, n(%) |  |  | 0.420 |
| No | 171 (91.0%) | 41 (87.2%) |  |
| Yes | 17 (9.04%) | 6 (12.8%) |  |
| WBC (×10^9^/L) | 3.99 (2.71) | 4.59 (3.55) | 0.283 |
| HB (g/L) | 86.3 (20.6) | 92.3 (26.8) | 0.152 |
| PLT (×10^9^/L) | 119 (87.1) | 128 (89.1) | 0.536 |
| Serum sodium (mmol/L) | 138 (4.35) | 139 (4.53) | 0.687 |
| ALT (U/L) | 68.1 (104) | 51.5 (72.3) | 0.202 |
| AST (U/L) | 92.7 (212) | 52.1 (55.9) | 0.021 |
| TG (mmol/L) | 2.88 (2.37) | 2.06 (1.03) | <0.001 |
| HDL (mmol/L) | 0.97 (0.50) | 1.04 (0.38) | 0.367 |
| LDH (U/L) | 522 (545) | 414 (279) | 0.060 |
| Serum ferritin (μg/L) | 5104 (9417) | 4784 (9661) | 0.839 |
| CRP (mg/L) | 23.9 (39.7) | 22.4 (32.9) | 0.785 |
| FIB (g/L) | 2.65 (1.54) | 45.8 (95.8) | 0.003 |
| PT (s) | 13.9 (3.22) | 9.41 (4.76) | <0.001 |
| TNF-α (pg/mL) | 11.0 (21.6) | 7.42 (15.6) | 0.193 |
| MAS secondary to SLE, n(%) |  |  | 0.819 |
| No | 94 (50.0%) | 25 (53.2%) |  |
| Yes | 94 (50.0%) | 22 (46.8%) |  |

**
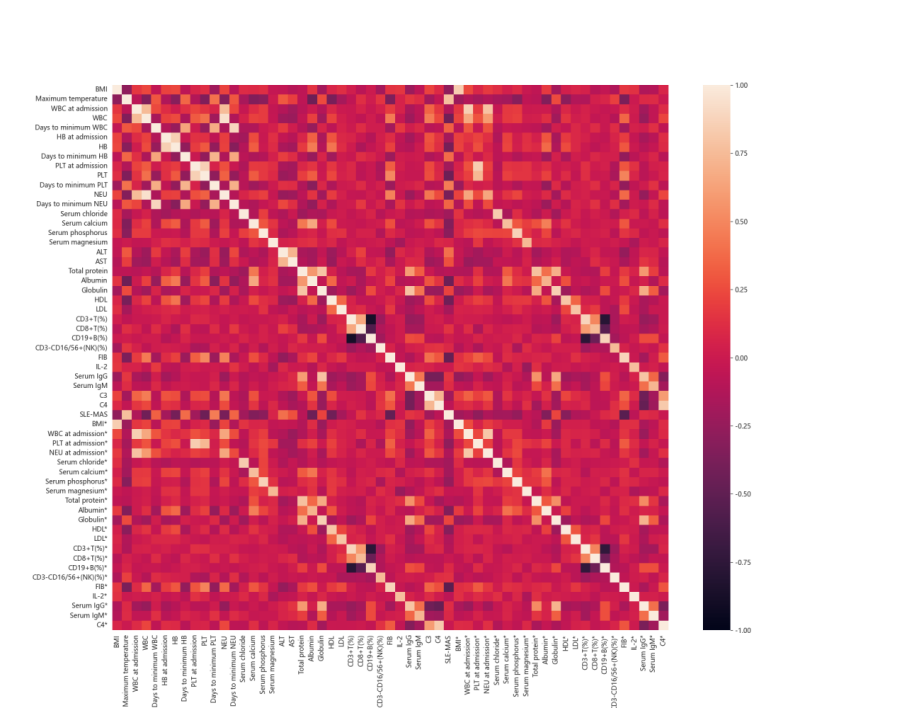
**

Supplement Figure S2 Pearson correlation coefficients of clinical features. The features marked with an asterisk (*) represent derived features calculated from the original feature and its corresponding normal range.

Supplement Table S 5 Variance inflation factors of clinical features. The features marked with an asterisk (*) represent derived features calculated from the original feature and its corresponding normal range.

| **Features** | **VIF** |
| --- | --- |
| Age | 4.680485052 |
| Gender | 4.865801757 |
| BMI | 16.7506478 |
| SLEDAI | 9.650390614 |
| Pulmonary infection | 5.422469604 |
| Immunodepressant | 4.653223269 |
| Pathogenic detection | 5.565920582 |
| Rash | 4.313671473 |
| Maximum temperature | 12.69111657 |
| Duration of fever | 6.425574439 |
| Joint pain | 7.601700236 |
| Hepatomegaly | 5.528344414 |
| Splenomegaly | 6.924487706 |
| Enlarged lymph node | 6.311915681 |
| Neuropsychiatric symptoms | 6.710821454 |
| WBC at admission | 24.48259474 |
| WBC | 44.07910539 |
| Days to minimum WBC | 29.19848215 |
| HB at admission | 32.39510974 |
| HB | 40.36861928 |
| Days to minimum HB | 11.56679378 |
| **Features** | **VIF** |
| PLT at admission | 36.55409145 |
| PLT | 26.81373222 |
| Days to minimum PLT | 12.47605872 |
| NEU at admission | 6.306252181 |
| NEU | 47.99633584 |
| Days to minimum NEU | 23.64925517 |
| TBIL | 12.18768681 |
| Serum sodium | 16.16244025 |
| Serum potassium | 7.104779002 |
| Serum chloride | 20.37307898 |
| Serum calcium | 16.31585769 |
| Serum phosphorus | 17.46501106 |
| Serum magnesium | 8.482067056 |
| ALP | 16.01825487 |
| ALT | 15.46220161 |
| AST | 11.10824325 |
| Total protein | 686.721428 |
| Albumin | 247.0513083 |
| Globulin | 537.4855612 |
| Serum creatinine | 5.744257298 |
| TG | 8.412173144 |
| HDL | 16.87862268 |
| LDL | 15.13751166 |
| LDH | 14.13176539 |
| Serum ferritin | 12.28815901 |
| ESR | 12.70588532 |
| **Features** | **VIF** |
| CRP | 14.97163993 |
| PCT | 12.81469619 |
| CD3+T(%) | 116.8369379 |
| CD4+T(%) | 40.28517827 |
| CD8+T(%) | 67.37242772 |
| CD19+B(%) | 110.7433609 |
| CD3-CD16/56+(NK)(%) | 22.9329256 |
| D-Dimer | 8.281356397 |
| FIB | 28.89000274 |
| APTT | 20.53751923 |
| PT | 18.74471605 |
| TT | 19.89470982 |
| Anti-dsDNA positive | 4.739154249 |
| IL-2 | 12.16017816 |
| IL-4 | 11.33257213 |
| IL-6 | 4.250305223 |
| IL-10 | 9.55344926 |
| TNF-α | 4.456642119 |
| INF-γ | 13.05268598 |
| Serum IgG | 14.10476941 |
| Serum IgA | 3.368016798 |
| Serum IgM | 10.22032782 |
| C3 | 11.03766849 |
| C4 | 19.80382453 |
| EBV-positive | 4.316664693 |
| Coxsackie virus B positive | 6.808085004 |
| **Features** | **VIF** |
| Enterovirus-positive | 5.798682527 |
| CMV-positive | 5.717425674 |
| Hemophagocytosis | 4.201833823 |
| Pericardial effusion/PEF | 5.935897976 |
| Pericardial effusion/PE | 8.703098711 |
| BMI* | 16.75490352 |
| WBC at admission* | 13.94585891 |
| HB at admission* | 10.10613999 |
| PLT at admission* | 20.00572171 |
| NEU at admission* | 11.48752928 |
| TBIL* | 6.543114089 |
| Serum sodium* | 5.349429469 |
| Serum potassium* | 10.741454 |
| Serum chloride* | 14.37209688 |
| Serum calcium* | 11.37587971 |
| Serum phosphorus* | 14.75968441 |
| Serum magnesium* | 8.991408502 |
| ALP* | 6.273042832 |
| ALT* | 8.522499119 |
| AST* | 12.29206333 |
| Total protein* | 9.779305468 |
| Albumin* | 7.143222291 |
| Globulin* | 20.48191852 |
| Serum creatinine* | 6.497020853 |
| TG* | 5.101188522 |
| HDL* | 14.0207344 |
| **Features** | **VIF** |
| LDL* | 11.70320207 |
| LDH* | 4.447376633 |
| Serum ferritin* | 5.174178801 |
| ESR* | 6.237514754 |
| CRP* | 5.393154289 |
| PCT* | 12.44332642 |
| CD3+T(%)* | 17.04588593 |
| CD8+T(%)* | 11.20428588 |
| CD19+B(%)* | 25.94407641 |
| CD3-CD16/56+(NK)(%)* | 8.360552476 |
| D-Dimer* | 6.909189605 |
| FIB* | 15.99992535 |
| APTT* | 11.32490019 |
| PT* | 10.32652703 |
| TT* | 7.099946771 |
| IL-2* | 8.340341019 |
| IL-4* | 9.828971159 |
| IL-6* | 5.942807139 |
| IL-10* | 6.093958697 |
| TNF-α* | 7.155991764 |
| INF-γ* | 6.004617088 |
| Serum IgG* | 16.22865319 |
| Serum IgA* | 5.255292782 |
| Serum IgM* | 8.870361426 |
| C3* | 8.151469226 |
| C4* | 16.79620846 |
| **Features** | **VIF** |
| ANA positive titers1:100 | 3015.503333 |
| ANA positive titers1:320 | 8047.680799 |
| ANA positive titers1:1000 | 3108.348262 |
| ANA positive titers1:3200 | 18832.9499 |
